# Refinement of the *HIVAN1* Susceptibility Locus on Chr. 3A1-A3 via Generation of Sub-Congenic Strains

**DOI:** 10.1371/journal.pone.0163860

**Published:** 2016-10-13

**Authors:** Natalia Papeta, Ami Patel, Vivette D. D’Agati, Ali G. Gharavi

**Affiliations:** 1 Department of Medicine, Columbia University, New York, New York, United States of America; 2 Department of Pathology, Columbia University, New York, New York, United States of America; Associate Professor, JAPAN

## Abstract

HIV-1 transgenic mice on the FVB/NJ background (TgFVB) represent a validated model of HIV-associated nephropathy (HIVAN). A major susceptibility locus, *HIVAN1*, was previously mapped to chromosome 3A1-A3 in a cross between TgFVB and CAST/EiJ (CAST) strains, and introgression of a 51.9 Mb segment encompassing *HIVAN1* from CAST into TgFVB resulted in accelerated development of nephropathy. We generated three sub-congenic strains carrying CAST alleles in the proximal or distal regions of the *HIVAN1* locus (Sub-II, 3.02–38.93 Mb; Sub-III, 38.45–55.1 Mb and Sub-IV, 47.7–55.1 Mb, build 38). At 5–10 weeks of age, histologic injury and proteinuria did not differ between HIV-1 transgenic Sub-II and TgFVB mice. In contrast, HIV-1 transgenic Sub-III and Sub-IV mice displayed up to 4.4 fold more histopathologic injury and 6-fold more albuminuria compared to TgFVB mice, similar in severity to the full-length congenic mice. The Sub-IV segment defines a maximal 7.4 Mb interval for *HIVAN1*, and encodes 31 protein coding genes: 15 genes have missense variants differentiating CAST from FVB, and 14 genes show differential renal expression. Of these, *Frem1*, *Foxo1*, and *Setd7* have been implicated in the pathogenesis of nephropathy. *HIVAN1* congenic kidneys are histologically normal without the HIV-1 transgene, yet their global transcriptome is enriched for molecular signatures of apoptosis, adenoviral infection, as well as genes repressed by histone H3 lysine 27 trimethylation, a histone modification associated with HIV-1 life cycle. These data refine *HIVAN1*to 7.4 Mb and identify latent molecular derangements that may predispose to nephropathy upon exposure to HIV-1.

## Introduction

HIV-1 associated nephropathy (HIVAN) is a major complication of HIV-1 infection, and results in end-stage renal disease without antiviral treatment [1, 2]. Clinically HIVAN manifests with proteinuria, and histologically it is characterized by collapsing focal and segmental glomerulosclerosis, microcystic tubular dilatation, and interstitial inflammation [3]. HIVAN arises due to HIV-1 induced dysregulation of podocytes, the glomerular epithelial cells that maintain the kidney filtration barrier [4–7]. HIVAN development has a strong genetic component both in humans and mouse models. In humans, HIVAN predominantly develops in individuals of African descent, and relatives of HIVAN patients have a higher incidence of end-stage renal disease [8]. Recently, studies have shown that susceptibility in humans is attributable to coding variants in *APOL1* that confer resistance to trypanosomiasis but increase susceptibility to kidney failure [9]. The mechanisms through which *APOL1* variants produce kidney injury are under active investigation [10, 11]. Although mice do not have an *APOL1* ortholog, transgenic expression of a replication deficient HIV-1plasmid that contains all the structural viral proteins except *Gag* and *Pol* reproduces characteristic lesions of HIVAN in the FVB/NJ genetic background (TgFVB strain) [4–6]. This finding indicates that perturbations in alternative biological pathways, in the absence of *APOL1*, can produce HIVAN in the mammalian kidney, and hence analysis of mouse models of HIVAN may inform the pathogenesis of human disease. The development of murine HIVAN is strain dependent, with the FVB/NJ as the most susceptible strain, while F1 hybrids of TgFVB with other inbred strains show variable susceptibility to disease [12–14]. We have used crosses between TgFVB and other inbred strains to map four nephropathy susceptibility loci (named *HIVAN1-4*) [12–14]. The *HIVAN1* susceptibility locus was previously mapped to chromosome 3A1-A3 in a cross between TgFVB and CAST/EiJ (CAST) strain. [12]. To confirm this locus, we previously generated a congenic strain, TgFVB-HIVAN1^CAST^, by introgressing a 51.9 Mb CAST interval encompassing the *HIVAN1* locus into the FVB genome [15]. While wild-type FVB-HIVAN1^CAST^ mice were phenotypically normal, HIV-1 transgenic counterparts developed early onset and more severe kidney disease by 6–8 weeks of age compared to TgFVB. This initial congenic interval contained over 300 protein coding genes, leaving open the possibility that multiple genes contribute to increased susceptibility to nephropathy. Here, we report generation and characterization of three sub-congenic strains that carry sub-regions of the original *HIVAN1* locus. These new *HIVAN1* sub-congenic strains allowed us to refine the *HIVAN1* locus to a maximum 7.4Mb interval, enabling detailed annotation of positional candidates and analysis of molecular pathways producing susceptibility to nephropathy.

## Materials and Methods

### Mouse strains and their genotypes

This study was carried out in accordance with the recommendations in the Guide for the Care and Use of Laboratory Animals of the National Institutes of Health. The protocol was approved by the IACUC committee at the Columbia University Medical Center. The mice were housed in a pathogen–free facility with 12 hour light cycle and were fed with a regular chow *ad libitum*.

**Fig 1:**
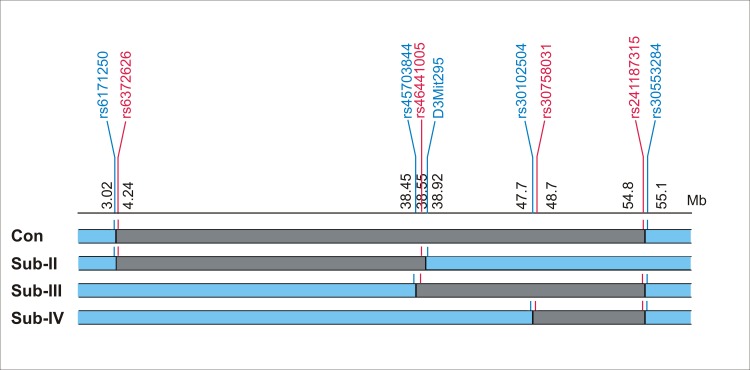
Map of the *HIVAN1* locus, congenic and sub-congenic regions. The rectangles depict the congenic and sub- congenic segments. The top line shows the position of the limiting markers in Mb (genome build 38p.3/mm10). The limiting markers with FVB genotypes are shown in blue, and those with CAST genotypes in red. The segments carrying CAST alleles are shown in grey. (Con = congenic strain)

The FVB/NJ mice were purchased from Jackson Laboratories. The HIV-1 transgenic mouse line TgN(pNL43d14)Lom 26 (TgFVB) on the inbred FVB/NJ genetic background and the TgFVB-HIVAN1^CAST^ congenic strain were previously described [15–17]. We backcrossed TgFVB-HIVAN1^CAST^ mice to FVB/NJ strain and identified recombinant mice to generate three sub-congenic lines containing smaller regions of the original congenic locus (**Fig 1**). The Tg-Sub-II-HIVAN1^CAST^ (Sub-II) strain carried CAST alleles between rs6372626 (4.25 Mb) and rs46441005 (38.55 Mb), delimited by FVB alleles at rs6171250 (3.02 Mb) and D3mit295 (38.93 Mb), defining a maximal interval size of 35.9 Mb. The Tg-Sub-III-HIVAN1^CAST^ (Sub-III) strain carried CAST alleles between rs46441005 (38.55 Mb) and rs241187315 (54.8 Mb) delimited by FVB alleles at rs45703844 (38.45Mb) and rs30553284 (55.1Mb), defining a maximal interval size of 16.65 Mb. The Tg-Sub-IV-HIVAN1^CAST^ (Sub-IV) strain carried CAST alleles between rs30758031 (48.7 Mb) and rs241187315 (54.8Mb), delimited by FVB alleles at rs30102504 (47.7Mb) and rs30553284 (55.1Mb) defining a maximal interval size of 7.4 Mb. The marker positions are indicated by genome build 38p.3. SNP IDs and annotation across the *HIVAN1* interval were obtained from the Mouse Phenome Database (http://phenome.jax.org/). The SNP annotations are presented in **S1 Table**.

Animals were euthanized (by CO_2_ asphyxiation followed by cervical dislocation) at 5–10 weeks of age and urine and kidneys were collected for phenotypic studies. Proteinuria and renal histology were compared between mice of differing genotypes at the *HIVAN1* locus.

### Evaluation of renal histopathology and albuminuria, and statistical analysis

**Fig 2:**
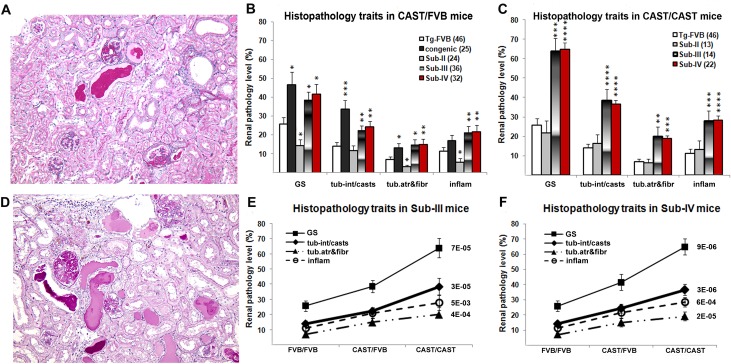
Comparison of renal histopathology between TgFVB, *HIVAN1* congenic and sub-congenic strains. **A &D.** Representative kidney histopathology in transgenic mice of Tg-FVB (**A**) and homozygous Sub-IV (**D**) genotypes. The images depict features of the histopathology scores that were close to the average for each group. Focal segmental and global glomerulosclerosis, tubular casts and focal interstitial inflammation were more severe in Sub-IV mice than in Tg-FVB mice (Periodic acid-Schiff, x200). **B& C.** Severity of renal histopathology in mice heterozygous (**B**) and homozygous (**C**) for CAST alleles across congenic segments. HIV-1 transgenic congenic, Sub-III and Sub-IV mice, but not Sub-II mice have statistically significant increased renal injury compared to TgFVB mice (* -p<0.05, ** -p<0.01, *** -p< 1x10^-3^, **** -p< 1x10^-4^., **E**&**F.** Additive effect of CAST alleles on renal injury in HIV-1 transgenic Sub-III (**E**) and Sub-IV (**F**) mice. The traits are shown as mean ± standard errors of mean. Panels **E** and **F** show genotypic comparisons for each trait by nonparametric test (Kruskal-Wallis one way ANOVA) with the associated p-values shown to the right of each curve.

Kidneys were formalin fixed and paraffin embedded, and 3 um sections were cut and stained with periodic-acid Schiff (PAS). Renal histology was scored independently by an investigator (VDD) blinded to genetic background, using a semi-quantitative scale. We scored the severity of glomerular injury (segmental and global glomerulosclerosis), tubulo-interstitial disease (tubular proteinaceous casts/ tubular cystic dilatation, tubular atrophy /interstitial fibrosis), and interstitial inflammation. The histology phenotypes were quantified according to the percent of glomeruli or percent cortical parenchyma affected in whole kidney cross-sections after visualization of at least 200 glomeruli. Representative images of characteristic HIVAN kidney histopathology features are shown in **Fig 2** and **S1 Fig**.

Albuminuria was quantitated in the urine of random subsets of mice of each genotype (4–10 mice in each group) and presented as albumin-to-creatinine ratio (ug/mg). Albumin and creatinine were measured with Albuwell M and Creatinine ELISA kits (Exocell, Philadelphia, PA).

Statistical analyses of proteinuria and histologic injury between TgFVB and TgFVB-HIVAN1^CAST^ strains were performed using Kruskal-Wallis Anova and two-sided Mann-Whitney nonparametric tests, using GraphPad Prism 7.01 software. P-values < 0.05 were considered significant.

### RNA isolation and microarray analysis

Total kidney RNA was isolated using trizol reagent (Invitrogen, Grand Island, NY), followed by treatment with DNaseI and clean-up using the RNeasy kit (QIAGEN) according to the protocols recommended by the manufacturers.

We performed microarray analysis with the Affymetrix ST 1.0 gene arrays (Santa Clara, CA). Total kidney RNA was extracted from 20 *HIVAN1* congenic mice (11 females / 9 males) and 19 FVBN/J littermates (10 females / 9 males). Sample preparation, labeling and hybridization were performed as per Affymetrix recommended protocol. Signal intensities were normalized using the RMA method. Differential gene expression was analyzed with two sided t-tests and corresponding False Discovery Rates (FDR) q-values were calculated. Pathway analysis was performed by computing overlap with two curated gene sets from the Molecular Signature Database (Canonical Pathways and Chemical and Genetic Perturbations, http://www.broadinstitute.org/gsea/msigdb/).We also cross-annotated the congenic kidney transcriptome with a recently described RNAseq transcriptome from murine FACS-sorted podocytes [18]. The transcriptome datasets are presented in **S2** and **S3 Tables**.

HIV interactions were queried from the NCBI HIV-1 Human Interaction Database.

## Results

### Characterization of HIVAN1 sub-congenic Sub-II, Sub-III and Sub-IV strains, carrying distal or proximal regions of the *HIVAN1* locus

The TgFVB-HIVAN1^CAST^ congenic strain carries a 51.9 Mb segment on Chr. 3 containing a susceptibility allele(s) for nephropathy from CAST, introgressed into the TgFVB genome [15]. To dissect the *HIVAN1* locus, we generated three sub-congenic strains carrying proximal or distal regions of the *HIVAN1* locus (Sub-II-HIVAN1^CAST^, Sub-III-HIVAN1^CAST^ and Sub-III-HIVAN1^CAST^, abbreviated as Sub-II, Sub-III and Sub-IV, respectively). The boundaries of the sub-congenic intervals are depicted in **Fig 1**. In the absence of the HIV-1 transgene, all congenic mice were phenotypically normal for up to 9 months of age and showed no histopathologic or biochemical evidence of nephropathy.

We first characterized HIV-1 transgenic mice heterozygous for each sub-congenic segment. The renal injury parameters did not differ between Tg-Sub-II^CAST/FVB^ and TgFVB (**Fig 2B**). In contrast, Tg-Sub-III-^CAST/FVB^ and Tg-Sub-IV^CAST/FVB^ mice showed a 1.5–2.4 fold increase in glomerulosclerosis, tubule-interstitial casts/cysts, tubular atrophy/interstitial fibrosis and inflammation (**Table 1** and **Fig 2**), and were comparable to the TgFVB-HIVAN1^CAST^ strain mice carrying the full congenic segment. This suggested that the HIVAN susceptibility gene(s) is encoded within the smaller Sub-IV interval.

**Table 1:** Renal pathology scores in TgFVB and HIV-1 transgenic congenic and sub-congenic strains.

	Phenotype	TgFVB	Congenic	Sub-II	Sub-III	Sub-IV
**CAST/FVB**	**age (weeks)**	7.3±.2	7.1±0.3	6.4±0.2	7.1±0.2	7.2±0.2
	**sex **	17M/29F	11M/14F	9M/15F	20M/16F	12M/20F
	**GS (%)**	26.4±3.6	46.7±6.6	14.3±3.2	38.9±4.4	41.6±5.3
	**Tub-int/casts (%)**	14.3±2	33.56±4.7	11.5±2.6	23.8±2.6	24.4±3
	**Tub. Atr. & Fibr(%)**	7.2±1.4	13.2±2.2	3±1	15.4±2.7	15±2.7
	**Inflam. (%)**	11.4±2.2	16.9±2.8	5.5±1.9	21.7±3.4	21.5±3.6
**CAST/CAST**	**age (weeks)**	7.3±.2	** **	6.5±0.2	7.4±0.5	6.7±0.3
	**sex**	17M/29F	-	5M/8F	7M/7F	9M/13F
	**GS (%)**	26.4±3.6	-	21.8±6.3	64.1±6.4	65±5.6
	**Tub-Int/casts (%)**	14.3±2	-	16.2±4.6	38.5±5.7	36±3.7
	**Tub. Atr. & Fibr(%)**	7.2±1.4	-	6.4±2	20.1±4.7	19.0±3.1
	**Inflam. (%)**	11.4±2.2	-	13.2±4.6	28.1±5	28.3±3.2

The histology phenotypes are expressed at percent of affected kidney segments after visualization of at least 200 glomeruli. GS = Percent glomeruli with sclerosis, Tub-Int/casts = Percent tubular interstitial cystic dilation/casts, Tub. Atr.&Fibr = Percent tubular atrophy and fibrosis, Inflam. = Percent of sections containing inflammatory infiltrates. Male (M) and female (F) distribution by group is also indicated. The trait values are shown as mean ± standard error of mean. Statistically significant differences between groups are indicated in **Fig 2**.

**Fig 3:**
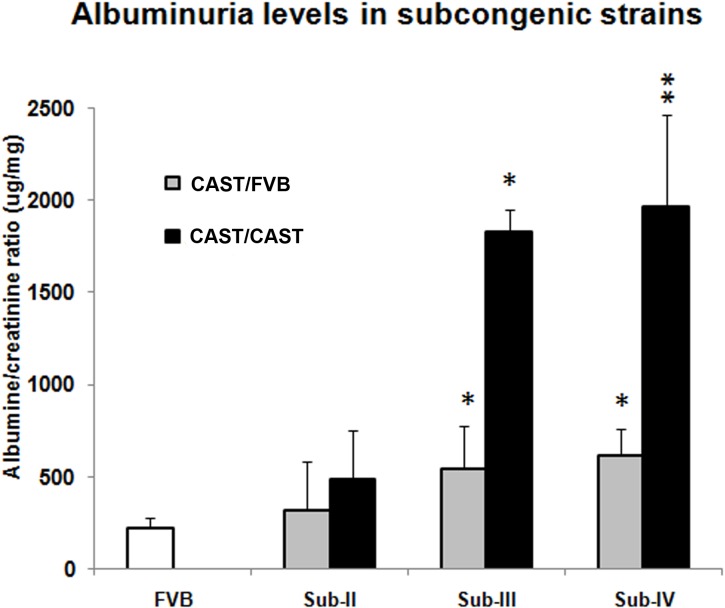
Albuminuria in TgFVB and the HIV-1 transgenic sub-congenic strains. Albuminuria is expressed as μg albumin/mg creatinine ratio. Statistically significant increase in albuminuria was observed in Sub-III and Sub-IV groups, but not in the Sub-II group when compared to TgFVB mice. (nonparametric P-value: *-p<0.05, **-p<0.01).

Next, we generated HIV-1 transgenic mice that were homozygous for each sub-congenic segment. Consistent with the phenotype of heterozygous congenic mice, Tg-Sub-II^CAST/CAST^ mice were indistinguishable from TgFVB. However, Tg-Sub-III^CAST/CAST^ and Tg-Sub-IV^CAST/CAST^ mice displayed advanced kidney disease, with 2.5–4.4 fold increase in severity across all histological parameters (p = 6x10^-7^–0.003 compared to TgFVB, **Table 1 and Fig 2C**). Thus the severity of kidney disease increased with the number of CAST alleles in Tg-Sub-III and Tg-Sub-IV mice, demonstrating an additive effect (**Fig 2E and 2F**, nonparametric p-value = 9x10^—6^5x10^-3^). Similar to the histopathology traits, albuminuria levels were not statistically different between Tg-Sub-II-^CAST/CAST^ and Tg-FVB mice, but were up to 6-fold higher in Tg-Sub-III and Tg-Sub-IV congenic mice, with an additive effect of CAST alleles (**Fig 3**).

In summary, only the Tg-Sub-III and Tg-Sub-IV mice showed increased severity of disease, capturing the phenotypic effect observed in the original congenic mice carrying the full *HIVAN1* congenic segment. Taken together, these data further confirm the *HIVAN1* locus and refine the susceptibility gene(s) to the 7.4Mb delimited by rs30102504 and rs30553284 within the Sub-IV region.

### Annotation and prioritization of genes within the Sub-IV locus

The Sub-IV locus spans 7.4Mb and encodes 31 RefSeq/UCSC annotated protein coding genes, 2 tRNAs, and 13 pseudogenes (**Table 2** and **S1 Table**). To identify putative sequence variants that may account for differential susceptibility to nephropathy, we compared the *Sub-IV* locus sequence between CAST/EiJ and FVB/NJ strains (Genome Build 38.p3). Consistent with the known genetic diversity between CAST/EiJ and laboratory-derived strains, all 31 RefSeq/UCSC annotated genes contained at least one coding and/or non-coding SNP differentiating the two strains. Among these, 15 genes had coding non-synonymous variants, but none harbored loss of function variants between the two strains (**S1 Table**). Missense variants in *Frem2*, *Mgarp* and *Rfxap* were predicted to be damaging by at least 1 program. There were multiple non-coding structural variants within this interval, including eight located within intronic regions of seven genes (**S1 Table**).

**Table 2:** List of candidate genes within the Sub-IV locus.

Gene	Position		No. of missense variants	Renal expression CAST vs. FVB		HIV Interactions	Molecular class
	start	end	CAST vs. FVB	Log2-fold	P-value		
*Pcdh18*	49743291	49757382	1	0.13	NS	-	Cadherin superfamily
*Slc7a11*	50364936	50499087	-	-0.12	NS	yes	Membrane transport protein
*Noct (Ccrn4l)*	51224447	51251654	-	-0.18	NS		Deadenylase
*Elf2*	51252720	51340644	2	0.03	NS		Transcription factor
*Mgarp (4930583H14Rik)*	51388412	51396547	1	-0.66	NS	-	Membrane protein (mitochondria)
***Ndufc1***	51405479	51408955	-	0.23	1.9E-04	-	Subunit of the NADH
***Naa15 (Narg1)***	51416016	51475985	-	0.12	0.007	-	Predicted N-acetyltransferase
*Rab33b*	51483966	51496228	-	0.00	NS	-	GTPase of the RAB family
Setd7*	51515318	51560823	1	-0.35	3.3E-05	yes	Arginine Methyltransferase
*Mgst2*	51559757	51567117	1	0.21	NS	-	Glutathione transferase
*Maml3*	51687320	52105085	1	-0.12	NS	-	DNA binding protein
Foxo1*	52268337	52350109	-	-0.11	0.02	-	Transcription factor
***Cog6***	52982123	53017223	2	0.12	2.E-04	-	Structural protein (Golgi complex)
***Lhfp***	53041547	53261679	-	-0.31	5.E-04	-	Integral membrane protein
***Nhlrc3***	53451996	53463258	-	-0.42	1.4E-11	-	Integral membrane protein
***Proser1(2810046L04Rik)***	53463817	53481755	2	-0.23	2.2E-06	-	Unknown
*Stoml3*	53488793	53507652	-	-0.07	NS	-	Integral membrane protein
Frem2*	53513938	53657912	8	-0.60	0.002	-	Extracellular matrix membrane protein (mutated in Fraser syndrome)
***Ufm1***	53853376	53863807	-	-0.18	2.2E-06		Unclassified (ubiquitin-like protein)
***Trpc4***	54156057	54318471	-	0.04	0.02	yes	Calcium Ion channel
***Postn***	54361096	54391041	1	-0.61	0.02	yes	Adhesion molecule
***Supt20 (D3Ertd300e)***	54692761	54728763	-	-0.20	1.1E-05	-	Transcription regulatory protein
*Exosc8*	54728679	54735364	2	-0.11	NS	-	Ribonuclease
***Alg5***	54735539	54749795	-	0.11	0.001	-	Glycosyltransferase
*Smad9*	54755457	54801741	-	0.05	NS	yes	A member of the SMAD family
*Rfxap*	54803115	54807791	3	0.04	NS	-	DNA binding protein
*Sertm1(6030405A18Rik)*	54897068	54915887	-	0.07	NS	-	Unclassified
*Ccna1*	55045469	55055330	-	-0.03	NS	yes	Cell cycle control protein
*Spg20*	55112074	55137332	2	0.04	NS	-	Unclassified
*Ccdc169 (A730037C10Rik)*	55137339	55175250	1	-0.01	NS	-	Unknown
*Sohlh2*	55182044	55209957	1	-0.04	NS	-	Transcription factor

Note: Only significant t-test p-values <0.05 are shown in the table. NS not significant.

*indicates genes implicated in nephropathy. The missense variants are listed in **S1 Table**.

Concurrently with generation of sub-congenic strain, we performed genome-wide expression profiling of whole kidneys from wild-type full congenic FVB-HIVAN1^CAST^ and FVB strains. We only profiled healthy wild-type kidneys because the profound histopathological lesions of HIV-1 transgenic mice introduce many secondary gene expression changes that can confound interpretation of transcriptomic data. We identified 327 differentially expressed transcripts between the two strains at a FDR q-value < 0. 1 (corresponding to a nominal *P*-value < 0.0014, **Table 2 and S1 Table**). As expected, the majority of the genes that were most differentially expressed were encoded within the congenic interval, indicating cis-eQTL effects. Within the Sub-IV locus,14 genes were differentially expressed between the two strains at a nominal p-value < 0.05. Three genes (*Frem2*, *Foxo1 and Setd7)* have been implicated in nephropathy [19–21]. In addition, 6 genes have an interaction with HIV-1 documented in the NCBI database (**Table 2**). Finally we cross-annotated our data with a recently published podocyte RNA-seq transcriptome dataset (**S2 Table**) [18]. Five genes located within the Sub-IV locus (*Ndufc1*, *Setd7*, *Ufm1*, *Alg5*, *Cog6*) are in the top 50^th^ percentile for podocyte-expressed transcripts and are also differentially expressed in congenic mice (**Table 2**). Of note, *Ndufc1*, encoding a subunit of the NADH dehydrogenase in mitochondria, is in the top 10^th^ percentile of podocyte expressed transcripts [18] and is also highly expressed in human kidney according to Genotype-Tissue Expression database (GTEx: http://www.gtexportal.org/). Complex I enables electron transfer from NADH to Coenzyme-Q_10_ and mutations affecting CoQ_10_ biosynthesis can cause nephrotic syndrome [22, 23]. Hence annotation of the Sub-IV locus identified a number of plausible candidates that require further investigation.

### Molecular perturbations in the renal transcriptome encoded outside the *HIVAN1* interval

To gain insight into pathways that are regulated downstream of the *HIVAN1* locus, we examined transcripts encoded outside the 51.9 Mb *HIVAN1*congenic interval. Although FVB and FVB-HIVAN1^CAST^ mice are genetically identical outside the *HIVAN1* interval, we identified 287 differentially expressed transcripts at FDR q-value <0.1 (corresponding to a nominal p-value of 0.0013), whereas only 34 transcripts would be expected to reach this significance level by chance. Because the two strains are genetically identical outside the *HIVAN1* interval, these expression differences are attributable to a primary genetic perturbation within the *HIVAN1* locus. Pathway analysis of all differentially expressed transcripts identified significant enrichment for multiple molecular signatures, including apoptosis induced by TRAIL, doxorubicin and serum deprivation (**Table 2** and **S1 Table**). In addition, we detect signatures of histone methylation, extracellular matrix components, and adenoviral infections. A number of these molecular signatures may be attributable to genetic perturbations within the *HIVAN1* locus. For example, the molecular signature for TRAIL-induced apoptosis is likely a consequence of a strong cis-eQTL for *Tnfsf10*, encoded within the *HIVAN1* locus, with the CAST allele associated with a nearly two-fold increased expression. *Tnfsf10* encodes TRAIL, a cytokine involved in induction of apoptosis in transformed and tumor cells. The molecular signature for extracellular matrix components is also noteworthy, because the *HIVAN1* Sub-IV interval contains *Frem2*, encoding a component of the extracellular matrix within the glomerular filtration barrier. The *HIVAN1* congenic mice have reduced expression of *Frem2*, which may account for reduced expression of multiple extracellular matrix components, such as *Col4a3* and *Col4a4*, encoded outside the *HIVAN1* locus. In addition, *HIVAN1* congenic kidneys harbor the signature of Polycomb target gene sets (histone H3 lysine 27 trimethylation), which marks repressed gene transcriptional programs observed in embryonic stem cells and poorly differentiated tumors [24]. Consistent with these data, the majority of these Polycomb targets show reduced expression in *HIVAN1* congenic kidneys. In addition, this histone modification is associated with HIV-1 latency and reactivation [25, 26]. Finally, analysis of whole kidney and the podocyte-enriched transcripts in *HIVAN1* mice revealed significant overlap with molecular signatures of viral infection, particularly adenovirus (**Table 3, Table B** in **S2 Table**) and **Table B** in **S3 Table**)). These data further suggest the presence of baseline molecular perturbations that may be magnified in the setting of HIV-1 infection.

**Table 3:** Pathway analysis of differentially expressed genes encoded outside the *HIVAN1* locus

Gene Set Name	# Genes in Overlap		p-value		FDRq-value
MARSON BOUND BY FOXP3 UNSTIMULATED	26		1.78E-11		8.39E-08
MIKKELSEN MEF HCP WITH H3K27ME3	17		7.30E-10		1.73E-06
GRAESSMANN APOPTOSIS BY DOXORUBICINDN	28		2.34E-09		2.76E-06
HAMAIA POPTOSIS VIA TRAIL UP	13		1.43E-06		8.44E-04
MEISSNER NPCHCP WITH H3K4ME2 AND H3K27ME3	10		2.68E-06		1.27E-03
GRAESSMANN APOPTOSIS BY SERUM DEPRIVATION UP	11		2.60E-05		6.81E-03
DORN ADENOVIRUS INFECTION 24HR DN	4		3.34E-05		7.25E-03
PILON KLF1T ARGETS DN	22		3.43E-05		7.25E-03
NABA MATRISOME	15		3.53E-05		7.25E-03
BENPORATHES WITH H3K27ME3	15		9.01E-05		1.25E-02
MIKKELSEN NPC HCP WITH H3K27ME3	8		1.10E-04		1.46E-02
MIKKELSEN ES ICP WITH H3K4ME3	11		2.62E-04		2.57E-02
PIDAVB3 INTEGRIN PATHWAY	4		2.96E-04		2.64E-02
DORN ADENOVIRUS INFECTION 12HR DN	3		3.68E-04		3.08E-02
NABA MATRISOME ASSOCIATED	11		3.91E-04		3.08E-02
DORNA DENOVIRUS INFECTION 32HR DN	3		6.05E-04		4.09E-02
DORN ADENOVIRUS INFECTION4 8HR DN	3		6.52E-04		4.22E-02
MEISSNER BRAIN HCP WITH H3K4ME3 AND H3K27ME3	13		6.69E-04		4.27E-02

Gene set names are from the Molecular signature database. Selected enriched pathways with FDR q-value <0.05 are shown. The full results of the pathway analyses are shown in **Tables A** and **B** in **S2 Table**.

## Discussion

HIVAN and other forms of collapsing glomerulopathy have a complex determination and result from environmental insults (e.g. viruses or drugs) [27] as well as host genetic lesions [9, 22, 23]. Although mice do not have an *APOL1* ortholog, the TgFVB mice recapitulates all of the clinical and molecular features of HIVAN [4–6], providing a model enabling for studying molecular mechanisms of glomerulosclerosis independent or downstream of *APOL1*. Murine susceptibility loci may also explain pathways leading to nephropathy in patients who do not harbor *APOL1* risk alleles.

We had previously generated a *HIVAN1* congenic mouse strain which carries a ~52 Mb segment from CAST in the FVB genome [15]. This strain did not show any spontaneous signs of renal disease, but in the presence of the HIV-1 transgene, showed increased severity of nephropathy under an additive genetic model. Because large congenic intervals may contain multiple linked genes that may together contribute to the association with disease severity, we further dissected the *HIVAN1* locus by generation of three new *HIVAN1* sub-congenic strains. The two congenic strains carrying the distal portion of the *HIVAN1* locus captured all the phenotypic severity of the original congenic strain, and refine *HIVAN1* to a 7.4 Mb interval within the Sub-IV region. These data indicated that that the *HIVAN1* QTL signal is not attributable to widely distant genes within the original interval.

Annotation of the remaining positional candidates identified several genes that may contribute to disease. Three positional candidates have been implicated in kidney disease. *Frem2* is expressed in adult glomeruli, collecting ducts and transiently expressed in nascent nephrons (tubule and podocyte epithelia) [28]. FREM2 is required for maintenance of the integrity of the skin epithelium *in utero*, for renal development and for the maintenance of renal epithelial structure in adult mice [19]. Mutations in the human ortholog cause Fraser syndrome, which features renal agenesis and cystic, dysplastic or hypoplastic kidneys. Although *Frem2* haploinsufficiency does not overtly affect nephrogenesis in mice, expression of *Frem2* in adult kidneys correlated with cyst formation in homozygous mutant mice, indicating that the gene is required for maintaining the differentiated state of renal epithelia [28]. The CAST strain harbors multiple linked non-synonymous variants in *Frem2*, and this gene is also differentially expressed in the congenic mouse kidney. This variation in *Frem2* sequence and expression likely accounts for perturbed expression of multiple matrix components, such as *Col4a3* or*Col4a4*, which are encoded outside the *HIVAN1* locus (**S1 Table**).

The transcription factor *Foxo1* has been implicated in progression of nephropathies of different etiology, including hypertensive and diabetic nephropathy [29]. A recent study showed that upregulation of *Foxo1* expression in the kidney by transduction with recombinant lentivirus ameliorated podocyte injury and reduced severity of the symptoms in diabetic rats [20]. *Foxo1*may participate in the pathogenesis of HIVAN via multiple biological mechanisms including in cell cycle regulation [30], oxidative stress response [31, 32] and inflammation pathways [33, 34].

The Sub-IV interval also encodes SETD7, which plays a prominent role in lysine methylation of histone and non-histone proteins and is an important regulator of different transcription factors, including p53 [35], E2 promoter-binding factor 1 (E2F1) [36], the islet β cell factor PDX1 [37], NF-kB and others [38]. SETD7 can affect cell proliferation and apoptosis via co-activation of E2F1, modification of Wnt signaling, or regulation of β-catenin stability [39]. SETD7 is also a co-activator of HIV-1 transcription, which could contribute to the development of HIVAN: binding of SETD7 to HIV-1 TAR RNA and monomethylation of the viral transactivator Tat enhances HIV transcription [40]. A recent study also reported that SETD7 expression is associated with the degree of fibrosis in patients with IgA and membranous nephropathy and inhibition of SETD7 suppressed renal fibrosis in unilateral ureteral obstruction mice [21].

*Ndufc1*, encoding a subunit of the NADH dehydrogenase (complex I) in mitochondria, is highly enriched in podocytes and was also overexpressed in *HIVAN1* congenic kidneys. Complex I enables electron transfer from NADH to Coenzyme-Q_10_ and mutations in the biosynthetic pathway for CoQ_10_ cause syndromic as well as isolated forms of nephrotic syndrome [22, 23]. However, *Ndufc1* is overexpressed in the congenic kidneys and together with the absence of perturbations of oxidative phosphorylation pathways, this reduces the likelihood that *Ndufc1*is the causal gene in the *HIVAN1* interval.

We had previously hypothesized that HIVAN susceptibility loci introduce moderate genetic lesions that are tolerated, but are unmasked in the presence of the HIV-1 gene product [13]. Consistent with this hypothesis, analysis of apparently healthy *HIVAN1* congenic kidneys, in the absence of HIV-1, demonstrated perturbations in many transcripts encoded outside the locus. Analysis of differentially expressed transcripts indicated overlap with multiple gene sets for apoptotic pathways and tissue matrix components. We also a signature for targets of Krüppel-like factor1, which belongs to a class of transcription factors that have been implicated in HIVAN and other forms of nephropathy [41–43]. Furthermore, we detected significant overlap with genes silenced Polycomb-group protein-mediated histone H3 lysine 27 trimethylation (H3K27me3). This chromatin modification is observed in embryonic stem cells and in poorly differentiated tumors [24]. Moreover, H3K27me3 is implicated in epigenetic silencing of HIV-1 long terminal repeats and regulation of viral latency [25, 26]. Recent data also indicate that the HIV-1 Tat protein, which activates host programs that augment HIV-1 transcription, preferentially binds to host transcription start sites enriched for H3K27me3 marks [44]. Interestingly, we also detected a molecular signature of adenoviral infection, potentially indicating latent perturbations that may enhance susceptibility to viral injury. These data suggest a complex interplay between viral and host histone modification, and susceptibility to nephropathy.

In summary, analysis of congenic lines identified a number of plausible candidates that can single-handedly or cooperatively contribute to increased susceptibility to nephropathy. Transcriptomic analyses also suggested that *HIVAN1* congenic kidneys may be poised for dysfunction, and exposure to appropriate triggers such as HIV-1 gene products may produce molecular decompensations that lead to overt kidney disease. The standard follow-up of these findings would involve generation of additional sub-congenic strains harboring smaller *HIVAN1* segments to pinpoint the causal allele(s). In addition, newer mouse strains such as the Collaborative Cross or the Diversity Outbred strains offer a high resolution map of mouse haplotypes and may aid in refinement of QTL intervals [45–47]. Most importantly, the availability of CRISPR/Cas technology now allows rapid introduction of CAST alleles into the FVB germline, enabling assessment of phenotypic consequences of candidate sequence variants [48]. The combination of these approaches is expected to accelerate the identification of causal alleles contributing to kidney disease in mouse models.

## Supporting Information

S1 Fig Representative kidney histology images showing HIVAN pathology features. **A**. A representative image from Tg-FVB shows focal segmental glomerulosclerosis, podocyte swelling, focal casts, proximal tubular protein resorption droplets and interstitial inflammation. (PAS, x400). **B-D**. Representative images from Sub-IV show (**B**) focal segmental and global glomerulosclerosis with adjacent large tubular casts (PAS x400), (**C**) extensive focal segmental glomerulosclerosis, focal interstitial fibrosis, interstitial inflammation and casts (PAS, x200) and (**D**) numerous proteinaceous casts (PAS, x200).
(TIF)

S1 Table Coding SNPs and intronic indels in the Sub-IV region. (XLSX)

S2 Table **Table A. Transcriptome in HIVAN1 congenic mice (CAST) vs. FVB mice. Table B. GSEA analysis of genes encoded outside the HIVAN1 locus and differentially expressed (at FDR<0.1) in HIVAN1 congenic mice vs. FVB/NJ mice. Table C. Transcripts in the Matrisome (M5889) and H3K27ME3 (M2019) gene sets.**
(XLSX)

S3 Table **Table A. HIVAN1 congenic mouse transcriptome cross- annotated for murine podocyte expression. Table B. GSEA analysis of podocyte enriched genes.** Top 10th percentile RPKM that are differentially expressed in HIVAN1 congenic mice vs. FVBN/J mice at FDR q-value <0.25 are shown.
(XLSX)

## References

[1] Rao TK, Filippone EJ, Nicastri AD, Landesman SH, Frank E, Chen CK, et al Associated focal and segmental glomerulosclerosis in the acquired immunodeficiency syndrome. The New England journal of medicine. 1984;310(11):669–73. 10.1056/NEJM198403153101101 . 6700641

[2] Selik RM, Byers RH Jr., Dworkin MS. Trends in diseases reported on U.S. death certificates that mentioned HIV infection, 1987–1999. J Acquir Immune Defic Syndr. 2002;29(4):378–87. Epub 2002/03/28. . 11917243 10.1097/00126334-200204010-00009

[3] D'Agati V, Appel GB. HIV infection and the kidney. J Am Soc Nephrol. 1997;8(1):138–52. . 9013459 10.1681/ASN.V81138

[4] Barisoni L, Bruggeman LA, Mundel P, D'Agati VD, Klotman PE. HIV-1 induces renal epithelial dedifferentiation in a transgenic model of HIV-associated nephropathy. Kidney international. 2000;58(1):173–81. 10.1046/j.1523-1755.2000.00152.x 10886562

[5] Winston JA, Bruggeman LA, Ross MD, Jacobson J, Ross L, D'Agati VD, et al Nephropathy and establishment of a renal reservoir of HIV type 1 during primary infection. The New England journal of medicine. 2001;344(26):1979–84. 10.1056/NEJM200106283442604 11430327

[6] He JC, Husain M, Sunamoto M, D'Agati VD, Klotman ME, Iyengar R, et al Nef stimulates proliferation of glomerular podocytes through activation of Src-dependent Stat3 and MAPK1,2 pathways. J Clin Invest. 2004;114(5):643–51. 10.1172/JCI21004 15343382 PMC514582

[7] D'Agati VD. Podocyte injury in focal segmental glomerulosclerosis: Lessons from animal models (a play in five acts). Kidney international. 2008;73(4):399–406. 10.1038/sj.ki.5002655 17989648

[8] Freedman BI, Soucie JM, Stone SM, Pegram S. Familial clustering of end-stage renal disease in blacks with HIV-associated nephropathy. Am J Kidney Dis. 1999;34(2):254–8. Epub 1999/08/04. 10.1053/AJKD03400254 S0272638699002747 [pii]. . 10430971

[9] Genovese G, Friedman DJ, Ross MD, Lecordier L, Uzureau P, Freedman BI, et al Association of trypanolytic ApoL1 variants with kidney disease in African Americans. Science. 2010;329(5993):841–5. Epub 2010/07/22. science.1193032 [pii] 10.1126/science.1193032 . 20647424 PMC2980843

[10] Nichols B, Jog P, Lee JH, Blackler D, Wilmot M, D'Agati V, et al Innate immunity pathways regulate the nephropathy gene Apolipoprotein L1. Kidney international. 2015;87(2):332–42. 10.1038/ki.2014.270 25100047 PMC4312530

[11] Olabisi OA, Zhang JY, VerPlank L, Zahler N, DiBartolo S 3rd, Heneghan JF, et al APOL1 kidney disease risk variants cause cytotoxicity by depleting cellular potassium and inducing stress-activated protein kinases. Proceedings of the National Academy of Sciences of the United States of America. 2016;113(4):830–7. 10.1073/pnas.1522913113 26699492 PMC4743809

[12] Gharavi AG, Ahmad T, Wong RD, Hooshyar R, Vaughn J, Oller S, et al Mapping a locus for susceptibility to HIV-1-associated nephropathy to mouse chromosome 3. Proc Natl Acad Sci U S A. 2004;101(8):2488–93. 10.1073/pnas.0308649100 14983036 PMC356977

[13] Papeta N, Chan KT, Prakash S, Martino J, Kiryluk K, Ballard D, et al Susceptibility loci for murine HIV-associated nephropathy encode trans-regulators of podocyte gene expression. J Clin Invest. 2009;119(5):1178–88. 10.1172/JCI37131 19381020 PMC2673856

[14] Prakash S, Papeta N, Sterken R, Zheng Z, Thomas RL, Wu Z, et al Identification of the nephropathy-susceptibility locus HIVAN4. J Am Soc Nephrol. 2011;22(8):1497–504. Epub 2011/07/26. 10.1681/ASN.2011020209 21784893 PMC3148704

[15] Chan KT, Papeta N, Martino J, Zheng Z, Frankel RZ, Klotman PE, et al Accelerated development of collapsing glomerulopathy in mice congenic for the HIVAN1 locus. Kidney international. 2009;75(4):366–72. 10.1038/ki.2008.625 19092797 PMC2753461

[16] Dickie P, Felser J, Eckhaus M, Bryant J, Silver J, Marinos N, et al HIV-associated nephropathy in transgenic mice expressing HIV-1 genes. Virology. 1991;185(1):109–19. . 1926769 10.1016/0042-6822(91)90759-5

[17] Kopp JB, Klotman ME, Adler SH, Bruggeman LA, Dickie P, Marinos NJ, et al Progressive glomerulosclerosis and enhanced renal accumulation of basement membrane components in mice transgenic for human immunodeficiency virus type 1 genes. Proc Natl Acad Sci U S A. 1992;89(5):1577–81. . 1542649 10.1073/pnas.89.5.1577 PMC48495

[18] Kann M, Bae E, Lenz MO, Li L, Trannguyen B, Schumacher VA, et al WT1 targets Gas1 to maintain nephron progenitor cells by modulating FGF signals. Development. 2015;142(7):1254–66. 10.1242/dev.119735 25804736 PMC4378252

[19] Jadeja S, Smyth I, Pitera JE, Taylor MS, van Haelst M, Bentley E, et al Identification of a new gene mutated in Fraser syndrome and mouse myelencephalic blebs. Nature genetics. 2005;37(5):520–5. 10.1038/ng1549 . 15838507

[20] Guo F, Zhang Y, Wang Q, Ren L, Zhou Y, Ma X, et al Effects of FoxO1 on podocyte injury in diabetic rats. Biochemical and biophysical research communications. 2015;466(2):260–6. 10.1016/j.bbrc.2015.09.024 . 26361145

[21] Sasaki K, Doi S, Nakashima A, Irifuku T, Yamada K, Kokoroishi K, et al Inhibition of SET Domain-Containing Lysine Methyltransferase 7/9 Ameliorates Renal Fibrosis. J Am Soc Nephrol. 2016;27(1):203–15. 10.1681/ASN.2014090850 . 26045091 PMC4696564

[22] Ashraf S, Gee HY, Woerner S, Xie LX, Vega-Warner V, Lovric S, et al ADCK4 mutations promote steroid-resistant nephrotic syndrome through CoQ10 biosynthesis disruption. J Clin Invest. 2013;123(12):5179–89. 10.1172/JCI69000 24270420 PMC3859425

[23] Sadowski CE, Lovric S, Ashraf S, Pabst WL, Gee HY, Kohl S, et al A single-gene cause in 29.5% of cases of steroid-resistant nephrotic syndrome. J Am Soc Nephrol. 2015;26(6):1279–89. 10.1681/ASN.2014050489 25349199 PMC4446877

[24] Ben-Porath I, Thomson MW, Carey VJ, Ge R, Bell GW, Regev A, et al An embryonic stem cell-like gene expression signature in poorly differentiated aggressive human tumors. Nature genetics. 2008;40(5):499–507. Epub 2008/04/30. 10.1038/ng.127 18443585 PMC2912221

[25] Friedman J, Cho WK, Chu CK, Keedy KS, Archin NM, Margolis DM, et al Epigenetic silencing of HIV-1 by the histone H3 lysine 27 methyltransferase enhancer of Zeste 2. Journal of virology. 2011;85(17):9078–89. Epub 2011/07/01. 10.1128/JVI.00836-11 21715480 PMC3165831

[26] Kim HG, Kim KC, Roh TY, Park J, Jung KM, Lee JS, et al Gene silencing in HIV-1 latency by polycomb repressive group. Virology journal. 2011;8:179 Epub 2011/04/19. 10.1186/1743-422X-8-179 21496352 PMC3094299

[27] Markowitz GS, Nasr SH, Stokes MB, D'Agati VD. Treatment with IFN-{alpha}, -{beta}, or -{gamma} is associated with collapsing focal segmental glomerulosclerosis. Clinical journal of the American Society of Nephrology: CJASN. 2010;5(4):607–15. 10.2215/CJN.07311009 20203164 PMC2849683

[28] Kerecuk L, Long DA, Ali Z, Anders C, Kolatsi-Joannou M, Scambler PJ, et al Expression of Fraser syndrome genes in normal and polycystic murine kidneys. Pediatric nephrology. 2012;27(6):991–8. 10.1007/s00467-012-2100-5 21993971 PMC3337421

[29] Luo WM, Kong J, Gong Y, Liu XQ, Yang RX, Zhao YX. Tongxinluo Protects against Hypertensive Kidney Injury in Spontaneously-Hypertensive Rats by Inhibiting Oxidative Stress and Activating Forkhead Box O1 Signaling. PloS one. 2015;10(12):e0145130 10.1371/journal.pone.0145130 . 26673167 PMC4686063

[30] Liu F, Ma XJ, Wang QZ, Zhao YY, Wu LN, Qin GJ. The effect of FoxO1 on the proliferation of rat mesangial cells under high glucose conditions. Nephrology, dialysis, transplantation: official publication of the European Dialysis and Transplant Association—European Renal Association. 2014;29(10):1879–87. 10.1093/ndt/gfu202 . 24914090

[31] Ponugoti B, Xu F, Zhang C, Tian C, Pacios S, Graves DT. FOXO1 promotes wound healing through the up-regulation of TGF-beta1 and prevention of oxidative stress. The Journal of cell biology. 2013;203(2):327–43. 10.1083/jcb.201305074 24145170 PMC3812981

[32] Furukawa-Hibi Y, Yoshida-Araki K, Ohta T, Ikeda K, Motoyama N. FOXO forkhead transcription factors induce G(2)-M checkpoint in response to oxidative stress. The Journal of biological chemistry. 2002;277(30):26729–32. 10.1074/jbc.C200256200 . 12048180

[33] Alikhani M, Alikhani Z, Graves DT. FOXO1 functions as a master switch that regulates gene expression necessary for tumor necrosis factor-induced fibroblast apoptosis. The Journal of biological chemistry. 2005;280(13):12096–102. 10.1074/jbc.M412171200 . 15632117

[34] Fan W, Morinaga H, Kim JJ, Bae E, Spann NJ, Heinz S, et al FoxO1 regulates Tlr4 inflammatory pathway signalling in macrophages. The EMBO journal. 2010;29(24):4223–36. 10.1038/emboj.2010.268 21045807 PMC3018786

[35] Liu X, Wang D, Zhao Y, Tu B, Zheng Z, Wang L, et al Methyltransferase Set7/9 regulates p53 activity by interacting with Sirtuin 1 (SIRT1). Proceedings of the National Academy of Sciences of the United States of America. 2011;108(5):1925–30. 10.1073/pnas.1019619108 21245319 PMC3033317

[36] Lezina L, Aksenova V, Ivanova T, Purmessur N, Antonov AV, Tentler D, et al KMTase Set7/9 is a critical regulator of E2F1 activity upon genotoxic stress. Cell death and differentiation. 2014;21(12):1889–99. 10.1038/cdd.2014.108 25124555 PMC4227146

[37] Maganti AV, Maier B, Tersey SA, Sampley ML, Mosley AL, Ozcan S, et al Transcriptional activity of the islet beta cell factor Pdx1 is augmented by lysine methylation catalyzed by the methyltransferase Set7/9. The Journal of biological chemistry. 2015;290(15):9812–22. 10.1074/jbc.M114.616219 25713082 PMC4392279

[38] Li Y, Reddy MA, Miao F, Shanmugam N, Yee JK, Hawkins D, et al Role of the histone H3 lysine 4 methyltransferase, SET7/9, in the regulation of NF-kappaB-dependent inflammatory genes. Relevance to diabetes and inflammation. The Journal of biological chemistry. 2008;283(39):26771–81. 10.1074/jbc.M802800200 18650421 PMC2546554

[39] Shen C, Wang D, Liu X, Gu B, Du Y, Wei FZ, et al SET7/9 regulates cancer cell proliferation by influencing beta-catenin stability. FASEB journal: official publication of the Federation of American Societies for Experimental Biology. 2015;29(10):4313–23. 10.1096/fj.15-273540 . 26116705

[40] Pagans S, Kauder SE, Kaehlcke K, Sakane N, Schroeder S, Dormeyer W, et al The Cellular lysine methyltransferase Set7/9-KMT7 binds HIV-1 TAR RNA, monomethylates the viral transactivator Tat, and enhances HIV transcription. Cell host & microbe. 2010;7(3):234–44. 10.1016/j.chom.2010.02.005 20227666 PMC2844784

[41] Mallipattu SK, Horne SJ, D'Agati V, Narla G, Liu R, Frohman MA, et al Kruppel-like factor 6 regulates mitochondrial function in the kidney. J Clin Invest. 2015;125(3):1347–61. 10.1172/JCI77084 25689250 PMC4362257

[42] Chen WC, Lin HH, Tang MJ. Matrix-Stiffness-Regulated Inverse Expression of Kruppel-Like Factor 5 and Kruppel-Like Factor 4 in the Pathogenesis of Renal Fibrosis. The American journal of pathology. 2015;185(9):2468–81. 10.1016/j.ajpath.2015.05.019 . 26212907

[43] Hayashi K, Sasamura H, Nakamura M, Sakamaki Y, Azegami T, Oguchi H, et al Renin-angiotensin blockade resets podocyte epigenome through Kruppel-like Factor 4 and attenuates proteinuria. Kidney international. 2015;88(4):745–53. 10.1038/ki.2015.178 . 26108068

[44] Marban C, Su T, Ferrari R, Li B, Vatakis D, Pellegrini M, et al Genome-wide binding map of the HIV-1 Tat protein to the human genome. PloS one. 2011;6(11):e26894 10.1371/journal.pone.0026894 22073215 PMC3208564

[45] Yalcin B, Nicod J, Bhomra A, Davidson S, Cleak J, Farinelli L, et al Commercially available outbred mice for genome-wide association studies. PLoS genetics. 2010;6(9):e1001085 Epub 2010/09/15. 10.1371/journal.pgen.1001085 20838427 PMC2932682

[46] Collaborative Cross C. The genome architecture of the Collaborative Cross mouse genetic reference population. Genetics. 2012;190(2):389–401. 10.1534/genetics.111.132639 22345608 PMC3276630

[47] Welsh CE, Miller DR, Manly KF, Wang J, McMillan L, Morahan G, et al Status and access to the Collaborative Cross population. Mammalian genome: official journal of the International Mammalian Genome Society. 2012;23(9–10):706–12. 10.1007/s00335-012-9410-6 22847377 PMC3463789

[48] Doudna JA, Charpentier E. Genome editing. The new frontier of genome engineering with CRISPR-Cas9. Science. 2014;346(6213):1258096 10.1126/science.1258096 . 25430774

