# Supplementary figures and images for: Refinement of the HIVAN1 Susceptibility Locus on Chr. 3A1-A3 via Generation of Sub-Congenic Strains
Source: PLoS One. 2016 Oct 13;11(10):e0163860. doi: 10.1371/journal.pone.0163860 (PMC5063463; doi:10.1371/journal.pone.0163860)

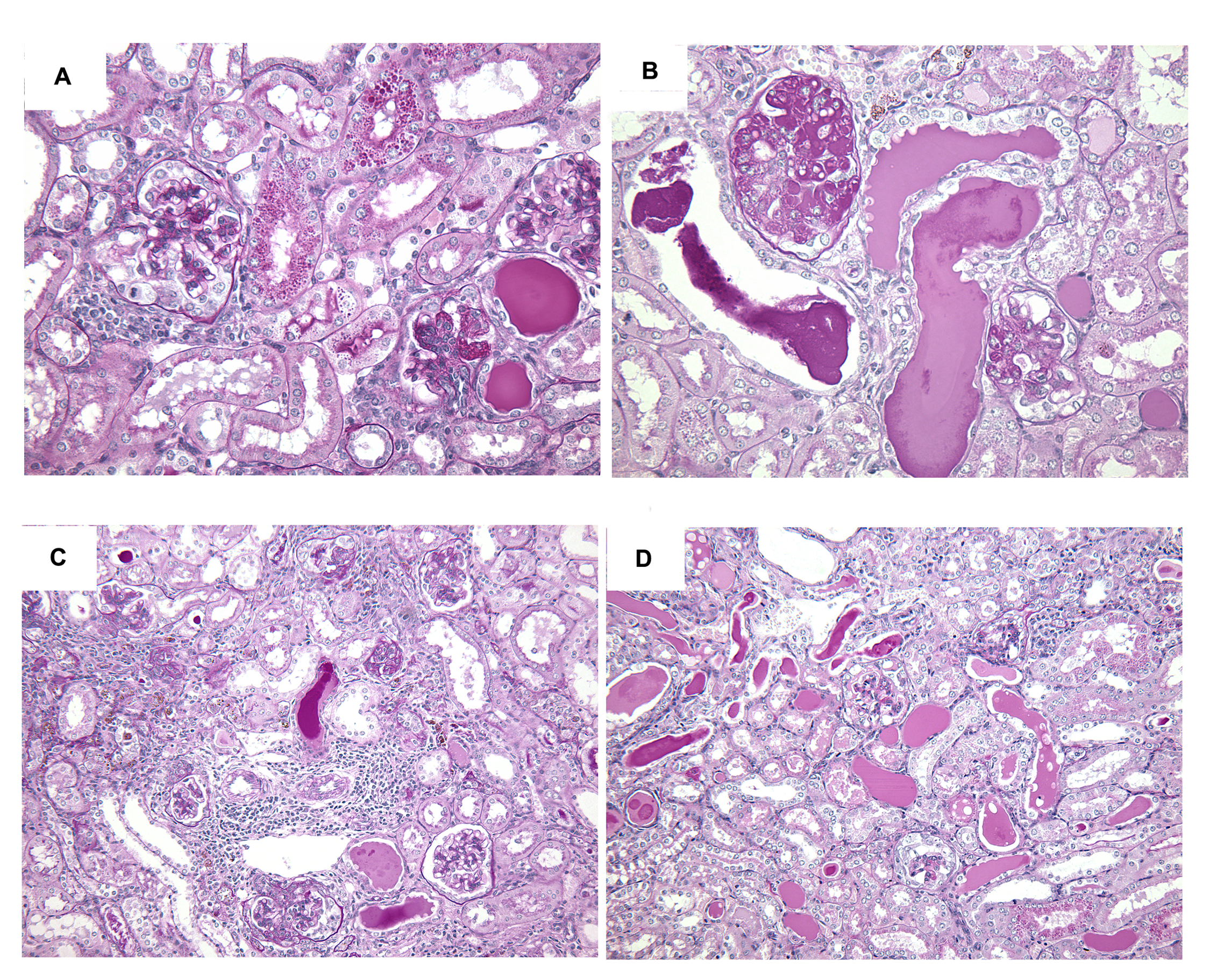

Supplement: S1 Fig — A. A representative image from Tg-FVB shows focal segmental glomerulosclerosis, podocyte swelling, focal casts, proximal tubular protein resorption droplets and interstitial inflammation. (PAS, x400). B-D. Representative images from Sub-IV show (B) focal segmental and global glomerulosclerosis with adjacent large tubular casts (PAS x400), (C) extensive focal segmental glomerulosclerosis, focal interstitial fibrosis, interstitial inflammation and casts (PAS, x200) and (D) numerous proteinaceous casts (PAS, x200). (TIF) [file pone.0163860.s001.tif]
